# Sharing is Caring: A Study of Food-Sharing Practices in Australian Early Childhood Education and Care Services

**DOI:** 10.3390/nu12010229

**Published:** 2020-01-16

**Authors:** Ruth Wallace, Karen Lombardi, Charlotte De Backer, Leesa Costello, Amanda Devine

**Affiliations:** 1 School of Medical & Health Sciences, Edith Cowan University, Perth, WA 6027, Australia; l.costello@ecu.edu.au (L.C.); a.devine@ecu.edu.au (A.D.); 2 Telethon Kids Institute, Perth, WA 6009, Australia; k.lombardi@telethonkids.org.au; 3 Department of Communication Studies, University of Antwerp, 2000 Antwerpen, Antwerp, Belgium; charlotte.debacker@uantwerpen.be

**Keywords:** food sharing, family-style mealtimes, physical, social and emotional development, Early Childhood Education and Care, Australia

## Abstract

Food connects people, and can significantly impact the physical, social and emotional development of young children. Food sharing and family-style mealtimes can support healthy eating practices and psychological well-being among young children, and carersother than family members, such as Early Childhood Education and Care staff, play an important role in the provision of these practices. Despite increasing numbers of Australian children attending Early Childhood Education and Care services, there is often reluctance among staff to promote such mealtime practices, to the detriment of children’s social and emotional development. The aim of this paper was to focus on the potential role of Early Childhood Education and Care services in facilitating food sharing and family-style mealtime practices in the earliest stages of the lifespan. A qualitative, netnographic approach was used, and data was collected as part of the broader ’Supporting Nutrition for Australian Childcare’ (SNAC) study, via online conversation threads, observations and qualitative interviews. Findings demonstrated that whilst many Early Childhood Education and Care services are committed to supporting food sharing and family-style mealtime practices, a number of barriers were reported. These included the perception that babies and toddlers could not participate in these practices, concerns about food hygiene and cross contamination of allergens, and negative parental influences on food sharing. In conclusion, this paper supports the practice of food sharing in Early Childhood Education and Care settings and calls for them to become embedded in everyday operations to support the physical, social and emotional development of Australia’s future generations.

## 1. Introduction

Globally, obesity in childhood has been cited as a neglected public health issue [1], resulting in serious short- and long-term risks to psychological and physiological health, such as low self-esteem [2], and higher risk of developing asthma, Type II diabetes and cardiovascular disease at a younger age [3]. One in five Australian children aged 2–4 years were reported as overweight/obese [4], increasing the risk of overweight/obesity in adulthood [5], and affecting both quality of life and life expectancy [6]. Furthermore, the health care costs associated with obesity in childhood are significantly higher than those for children of a healthy weight [7]. In recent decades, attempts to reduce the rates of overweight/obesity, such as weight loss interventions, have lacked success [8]. As such, there has been a refocus on preventing weight gain; the childhood years represent an important part of the life cycle where healthy practices, such as good nutrition, can be embedded to prevent poorer health outcomes [8].

The overweight/obesity problem affecting Australian children is influenced by “a complex interplay of individual, environmental and societal factors… including food and nutrition… and the obesogenic environment” [4]. Optimal nutrition in the first 1000 days of life is associated with improved academic outcomes for children [9], and reduced risk of neurodegeneration in later life [10]. The obesogenic environment includes the various physical settings that people interact with daily [4], and for young children, an important setting is the Early Childhood Education and Care (ECEC) sector (formally known as child care).

In Australia, ECEC services play a significant role in the well-being of children and their families, and changes to family structure, gender roles and the need for economic security have increased the demand for ECEC in recent years [11]. Currently, more than 1.35 million Australian children (44% of children aged 0–5 years) attend ECEC for on average 30 hours or 3 days per week, although some may attend for upwards of 45 h/week [12]. Children attending ECEC are likely to be involved with three mealtime experiences each day. Therefore, this environment has enormous potential to influence children’s eating habits and health outcomes into adulthood [13]. Global research has indicated, however, that ECEC settings do not harness such opportunities [14,15,16]. In fact, attendance at ECEC is associated with increased risk of overweight/obesity in childhood [17,18], especially if healthy eating and physical activity are not promoted, or lower quality standards exist [19].

The food environment, including limited access to healthy food choices also plays a significant role in childhood overweight/obesity [4], although very young children do not interact autonomously with this environment; more so it is the “nutritional gatekeepers… parents, grandparents and other caretakers (who) shape their nutritional ecosystem” [8]. One example of a health promoting approach to maintaining a healthy weight among young children that utilises the food environment is the practice of family-style mealtimes, i.e., sharing food at mealtimes. When families share meals, they also share time and conversations with each other, providing opportunities for moral socialization and order, i.e., who eats before, after, or at the same time as others, and especially rules about the social, or fair, distribution of food [20]. Studies have shown that children who frequently participated in food sharing at family mealtimes grew up to be more altruistic adults [21], suggesting this act of sharing a meal is about sharing respect, fairness and caring for each other. Family-style mealtimes allow children to recognise internal hunger cues and develop social skills [22] and are known to promote the intake of vegetables [23]. Moreover, mealtime environments that facilitate family communication and interaction appear to have a strong connection to mental health [24] and may also play a more important role in developing a healthy lifelong relationship to food overall. A meta-analysis of family-style dining frequency reported a “significant relationship between frequent family meals and better nutritional health” [8].

The many benefits for young children of enjoying food-sharing practices as part of a family mealtime, suggest this practice should not be restricted to a family context, as benefits can also be reaped from meals consumed elsewhere [25]. Studies have indicated that the benefits of sharing meals may also result from the presence of ‘other significant carers’ at family-style mealtimes, such as staff at ECEC services [26]. Therefore, a capacity building strategy for ECEC staff to ensure children’s health and wellness could be to promote family-style mealtimes, which include elements of food sharing.

For the purposes of this paper, a ‘family-style’ mealtime in ECEC is defined by: (a) children self-serving food *; (b) staff sitting with children at mealtimes; (c) staff eating the same foods as children*; (d) staff talking informally about healthy foods with children; (e) children being encouraged to try new foods; and (f) staff helping children to determine hunger before offering seconds [27,28]; (* denotes elements of the ‘family-style’ mealtime that involve sharing food). Promoting an active adult presence in the child mealtime environment is recognised as best practice in Australian and international ECEC settings [28,29] and reported as an ‘evidence-based practice” that supported conversations between children and educators, role-modelling “how to eat” and “interacting like a family” [30]. Such environments support the development of children’s self-esteem and are commonplace in Nordic ECEC settings where “pedagogic mealtimes” are promoted [26], based on the premise that role modelling is more powerful tool to teach young children about healthy eating than verbal messages alone [31].

Despite the many positive attributes of family-style dining, typically characterised by food sharing, such practices are not routine in ECEC settings globally [32] due to staff concerns about mess, hygiene and cross-contamination for children with allergies [33]. A US study reported ECEC staff having concerns about children serving themselves too much or too little food, and perceived conflict between the practice and guidelines [34]. Notwithstanding, a US study found that in ECEC services, staff were implementing at least one or two of the ‘best practice’ strategies recommended [29]. They reported that when staff simply sat with children at mealtimes and ate the same food, this resulted in children consuming more vegetables. In Sweden, an “ambition to integrate food into the pedagogic environment” is reported [35]—that is, discussions about healthy eating are embedded in usual practice, and children are exposed to healthy food choices that influence long-term eating habits. Similarly, a Canadian study describes mealtime discussions between ECEC staff and children as a “vehicle for teaching healthy eating” allowing for “a natural conversation” about food choices [36]. These studies reinforce the notion that when children eat in the presence of a ‘significant other’ (i.e., ECEC staff), it subsequently contributes to a healthier diet, alongside a range of other benefits, such as reduced incidences of food neophobia [37]. Ultimately, as food preferences and practices are initiated at a young age, ECEC settings are important environments where healthy relationships with food can be developed [38].

This paper presents qualitative data collected as part of a broader Australian studySupporting Nutrition for Australian Childcare (SNAC)—an online repository providing nutrition resources and discussion forums designed to support ECEC staff provide healthy eating environments. The site launched in 2013 and continues to support more than 2900 members across the Australian ECEC community [39].

This paper provides a narrative about the family-style dining experiences of SNAC members, highlighting enablers and barriers to this best-practice mealtime environment in ECEC settings. These barriers will be discussed alongside recommendations to overcome issues that hinder ECEC food-sharing practices. By doing so, this paper advocates that ‘sharing is caring’, whereby food is shared among and between children and their carers’, ultimately supporting optimal physical, social and emotional development.

## 2. Materials and Methods 

The SNAC study was conducted using a qualitative, netnographic methodology, developed by Robert Kozinets in the 1990s, which he recently refined in 2015, and characterised by data co-created by participants online [40]. The five unique steps of netnography described by Kozinets [41]—entrée, data gathering and analysis, trustworthiness, ethical research and feedback opportunities—were applied to ensure qualitative rigour; their application in this study is described next.

The first netnographic step of **entrée** is usually used to identify a suitable online community to study, though assessing relevancy, activity and interactivity of the membership [41]. In the case of SNAC, however, the purpose of the study was to develop a new community. Thus, the goals of the entrée stage—to build trust, rapport and acceptance within the new community—were essential to ensure appropriate audiences were targeted, enabling the co-creation of a culture attractive to intended participants [41]. Unique elicitation strategies, e.g., using professionally generated content to create a website attractive to new members and creating an impression of an active and successful website, were employed to drive participation and build the critical mass required to ensure community success [42]. 

To ensure the relevant community of interest were targeted [41], a purposive sampling technique was adopted and from a sampling frame of all licensed centre-based ECEC services, staff were emailed an invitation to participate. Informed consent was obtained from participants as they registered for the online community, and access granted only once explicit consent was provided. 

The second netnographic step, data gathering and analysis is a vital component that relies on communication within and between community members. These communications can occur in many different forms but, essentially, they should be between and within the individual community members, rather than the website [41]. Multiple data collection strategies are recommended, and were implemented, to ensure rigour, ensuring multiple participant perspectives were gathered [41]. 

Twelve months post-launch (2014), the SNAC community had a membership of 1045 members, with more than 56,000 page views. Key to the netnographic approach adopted, conversation threads (*n* = 183) and posts (*n* = 287) resulted in 1179 comments being made on the SNAC forums; these were captured for analysis, together with spontaneous participant observations gathered during researcher visits to ECEC services, and notes of email and telephone conversations. In addition, semi-structured interviews (*n* = 42) were conducted with individual SNAC members approximately 12 months post-launch, either face to face or by telephone, expediting the collection of rich, descriptive data, representing participant viewpoints and providing a flexible approach to collecting unexpected data [43]. 

The combination of data collection methods (Table 1) at different time points enabled data to be collected from differing perspectives and contexts, a triangulation strategy implemented to address credibility [44], ensuring strong, reliable findings, reduced bias and enriched interpretations [45].

NVivo (Version 10) was used to integrate data into one rational pattern for analysis, observing the key netnographic concept of coherence and enabling a unified, coherent interpretation of results, thus assuring confidence in their quality [41]. The analysis of data from the entire study was conducted by the first author who first deductively identified emerging themes that were descriptively coded, based on the study’s overarching research questions. 

In keeping with the netnographic methodology underpinning the broader SNAC study, the posting of questions and articles on the discussion boards occurred organically—that is, in response to events occurring in the ECEC space, or by queries made by the SNAC members themselves, thus assuring the co-creation of content [42].

Whilst many themes emerged during the data analysis phase, this paper provides a snapshot of the discussions between SNAC members and the researcher, specifically in relation to family-style mealtime environments and food-sharing issues experienced at ECEC services. The data informing this paper was gleaned from the interviews (Table 1) and posts made on the discussion boards by participants in response to the articles/themes identified in Table 2, and was summarised according to these headings:

The third netnographic step, trustworthiness, in qualitative terms, is usually measured in terms of transferability, credibility, dependability, confirmability and authenticity. In this paper, trustworthiness is demonstrated as a measure of quality enriched by a number of specific netnographic practices, interwoven through the original study and this paper (Table 3).

Kozinets [41] outlines the fourth netnographic step as ethical research, that involving human subjects as determined by ethics codes. Subsequently, as new SNAC members registered on the website, explicit informed consent was obtained. Member identities were protected, and pseudonyms used when reporting data. Ethics approval was granted by Edith Cowan University’s Human Research Ethics Committee (#8727). 

In the fifth netnographic step, Kozinets [41] suggests members should be provided with opportunities for feedback. When the SNAC community reached critical mass and appeared to be viable, members had the opportunity to co-create content online [40] and learn from each other. This community-created content was developed into specific resources, which were checked by participants to ensure their needs were met and was relevant, before being assimilated into more formal site features, such as factsheets and lesson plans.

## 3. Results/Discussion

This paper aimed to provide a narrative about the family-style dining experiences of SNAC members, and the enablers and barriers that affect the provision of such experiences in ECEC settings. The following findings demonstrate some ECEC services are committed to food-sharing practices and family-style mealtimes, whilst highlighting the obstacles reported that seemingly prevent these practices for some. 

At the time of data collection, there were 1045 SNAC members. The majority were female (98%), aged >36 years (75%), holding senior roles such as manager or director (60%), and working in city-based ECEC services (65%). 

### 3.1. Food Sharing Is Supported by Australian ECEC Services 

Participants were asked about mealtime practices in their own services, and responses illustrated goodwill towards and willingness to adopt food-sharing practices, such as children and ECEC staff eating together, and family-style dining. Katie, for example, was particularly optimistic, describing how the whole centre enjoyed such experiences: “At our centre we love to eat together—the whole centre if possible, so that siblings can sit with each other and we can all have a good old chinwag while we eat. It’s a very social occasion for us all (educators too). The kids serve their own meals and help themselves to more whilst the educators guide the conversation to discussions about healthy eating, sometimes and always foods, where things grow, how things are made, ingredients and shopping. It makes for a really enjoyable time” (Katie, Director).

On the SNAC forum, Bonnie was quick to agree with Katie’s remarks and noted this practice also fulfilled families’ wishes, and perhaps mirrored home mealtime environments for some: “I agree with Katie’s comments about set meals and everyone sitting together—following the families’ practices and wishes. I have always found that if all children come together for meal times they are more likely to sit longer and talk to the children at their table rather than jump up as soon as they have finished eating” (Bonnie, Director).

Similar mealtime practices were described by Janine, the director of a university-based ECEC centre, when children were allowed to serve themselves and choose their own foods from a buffet-style selection: “Here at Chase Childcare centre we have a Buffet Day and it is without a doubt the most popular day. Our children have an assortment of baked vegetables/steamed in season vegetables/salad i.e., Grated Carrot, Cherry Tomato, celery sticks just to name a few with the choice of Roast Veal or Tofu served with a little dinner roll… the idea is the children serve themselves with help and encouragement from our educators and it’s amazing how healthy and how much they eat when the choice is in their hands. It’s run like a Buffet in a restaurant so gorgeous to see the excitement” (Janine, Director).

These examples demonstrated that ECEC staff believed children enjoyed these experiences of sharing and self-serving food and reported how readily they selected healthy foods when given autonomy about food choices. A study by Shim and colleagues [46] also reported that allowing children to self-serve at mealtimes positively impacted subsequent vegetable intake. Furthermore, ECEC staff believed it important that children had autonomy over their food choices, as they observed these children were more willing to try new foods [33]. 

Other participants reported that whilst children are not necessarily serving themselves, they do sit together for their meals, and this time is utilised as an opportunity to discuss the cultural and social aspects of mealtimes: “Our children always sit at the tables together for meals… We believe eating together and talking about the meal is just as important as food itself. We discuss how food is enjoyed by different cultures—what tools are used (cutlery, chopsticks, hands)—how food is presented—and the roles people play in preparing the food” (Betty, Director).

Again, this is an encouraging finding, as the link between individual and social food habits established in early childhood is well-known [47]. Children’s social and emotional development occurs within a cultural context, so opportunities to experience and discuss other cultures promotes this development by guiding appropriate behaviours and promoting a sense of self [48]. The concept of culture was addressed by a centre director talking about activities for Harmony Week: “… sometimes it isn’t the food itself that is important for cultural inclusion, it’s how it is shared and eaten. I work with educators who have different cultural backgrounds and they regularly provide food for their children which is representative of their culture—but they don’t always talk with children about traditions for eating food, e.g., with fingers, which hand to use etc.” (Cassie, Director).

Whilst the cultural significance of food, and how it is eaten and shared, is important for children’s learning, some ECEC services deem children eating food with their fingers as impossible given the strict food hygiene and handling standards legislating practice at ECEC services [49], as discussed later in this paper.

Although the concept of food sharing and family-style mealtimes was supported by participants, some resistance was noted: “Some of them [directors/cooks] were keen to have the children self-serve… in other places it was ‘nuh, we’ll stand at the counter and hand it to them’… I think if children are involved in [self-serving food] as much as possible, for example, they can take a smaller or larger serve, they’ve taken responsibility for taking it… and can be coached to take responsibility for eating what they’ve taken, and then that’s a sort of respecting and valuing of the whole experience” (Betty, Director).

In spite of other staff members who were reluctant to engage with food-sharing practices, Betty explained her willingness to offer support as she believed these practices encouraged children to ‘take responsibility’, thus learning expected patterns of behaviour and developing the ability to manage and control their behaviour within these expectations [48]. However, the child’s age may influence the extent to which they are allowed or encouraged to engage with family-style dining.

### 3.2. Food-Sharing Options Depend on the Age of the Child

A Canadian study describes ECEC staff resisting food-sharing practices because they believed children were too young to manage this ‘relaxed’ mealtime environment [33]. Similarly, a Norwegian study reports that ECEC staff tend to have low expectations of young children’s ability and agency to cope with day-to-day situations [50], such as shared mealtimes. Katie (a director) provided her perspective about progressive mealtimes; a practice which typically involves food sharing and allows older children to approach the dining table to eat when hungry, rather than at a prescribed time: “A lot of centres implement progressive meals but we only really find it successful with the older group (3–5 years). The younger children dive at the table as soon as they realise it’s time to eat!! I have seen one centre actually take half a group of toddlers outside while they feed the other half then change over. This is not what progressive meals is about. When staff are still deciding when the children will eat and not allowing them to decide themselves. This is why we just let the younger ones eat all together if they approach the tables. The whole progressive mealtime does have its share of problems… it is still very adult-oriented in many ways”.

However, differing opinions about the appropriate age for children to engage in food-sharing practices were noted. For example, a food coordinator described how even younger children were actively encouraged to serve themselves: “I used to work in long day care centres where I promoted the right of all children from 2 years up to be able to choose their own food and serve themselves at mealtimes—not just when selecting fruit from a platter. We consistently found that even very small children can learn to serve themselves and to control their portion sizes… worked really well though and even fussy eaters ended up eating more than they normally would” (Carol).

Whilst ECEC staff concerns about younger children engaging in food sharing are understandable, it appears that infants and toddlers who were not exposed to these practices are missing opportunities to develop their social and emotional skills. A possible solution for these younger children to participate could be to introduce certain elements of baby-led weaning, a complementary feeding practice where infants are provided with a choice of texturally appropriate foods, for example, chunks of softened vegetables, and are encouraged to self-feed, as opposed to being spoon-fed by an adult [51]. Such practices, if adopted by ECEC services, may provide more opportunities for babies and toddlers to participate in shared meals and positive mealtime experiences. Furthermore, recent research has suggested that baby-led weaning may help reduce the risk of obesity [52].

Although there is considerable support for food sharing and family-style mealtimes at Australian ECEC services, other SNAC members indicated obstacles to the implementation of these practices. These are discussed in the following section.

### 3.3. Issues Affecting Food Sharing and Family-Style Meals

One of the main barriers to food sharing reported was the issue of food hygiene and food safety. For example, an educator stated that children would not use tongs when serving food, thus increasing [she believed] the risk of cross-contamination of bacteria: “… but practically, you can’t do food, because you’ve got the hygiene side of it, then you wouldn’t have them using tongs ever, they’d be nicking in, you’d have food left all over the place, you have sticky fingers and hands, you’d have, you know, sharing it’s just a minefield” (Karen).

Karen’s concerns are valid, as Australian ECEC services are strictly mandated to protect children from infectious diseases, as well as other threats to their health. Comprehensive government guidelines: ‘*Staying Healthy: Preventing infectious diseases in early childhood education and care services*’, [49] stipulate the following: “If children are sharing food from a common bowl or plate, make sure they understand that they need to use tongs, spoons or other appropriate utensils to take the food they want to eat. Remind them that they cannot touch food that is being shared because this can spread germs that might make them or other children ill. This is why it is important to use utensils, not your hands, when taking food from a common bowl or plate”.

Likewise, a similar Australian study reported ECEC staff discouraging children from using their fingers to serve themselves and share food [53]. Whilst these guidelines take a pragmatic, biomedical approach to food hygiene, they do not account for different cultural practices which children might engage in at home. Cherise described a scenario highlighting the limitations of the ECEC hygiene legislation: “I have even had a situation where an educator had a child attending who would normally eat with his hands at home, but she [the educator] was encouraging him to eat with utensils as she felt she ‘had to’ due to hygiene practices and because she didn’t want the other children to copy” (Cherise, FDC coordinator).

By encouraging this child to change his usual eating habits to which he was culturally attuned, this educator, although acting with best intentions, might have undermined the child’s sense of inclusion and engagement with that particular ECEC community. BeYou [54], the key Australian organisation supporting young children’s mental health, stated that: ECEC services can also support children and families from diverse backgrounds by promoting understanding of and mutual respect for diversity. Children benefit when ECEC services do this because it creates an inclusive environment where everyone can participate and feel connected. A strong sense of belonging helps children understand and appreciate differences in themselves and others, which ultimately benefits their mental health and well-being.

Other practices intended to minimise cross-contamination were also described, such as the birthday celebrations with a ‘fake’ cake to avoid the issue of spit: “We have a “fake” cake we pull out on birthday celebrations (more to address spit during the blowing out of candles). This then leaves us free to have the celebration food” (Marnie, Educator).

Marnie’s concerns about spit contamination are understandable—probably because of the social vulgarities associated with spit—but it was her creative solution to this problem which resonated with other SNAC members. In response to Marnie’s post, Kirsty shared a similarly creative solution: “I noticed the comment about spit on the birthday cake. I have always placed ‘Gladwrap’ [cling film] over the cake then place the candles on top through the ‘Gladwrap’. Children can still blow out the candles without spitting all over the cake then the candles and the “Gladwrap’ are removed to cut it into slices… easy peasy” (Kirsty, Director).

Australian government guidelines about birthday cakes do not include the use of ‘Gladwrap’ as described above, but recommend children are provided with individual cupcakes [49], unsupportive of the concept of food sharing, and undermining the collective and positive connotations associated with such birthday rituals [53]. 

Several studies have reported ECEC staff concerns about food sharing in relation to food hygiene and food safety. For example, Canadian studies by Lynch and Batal [33] and Lynch [36], and an Australian study [53] noted ECEC staff were apprehensive about supporting food-sharing practices because of concerns about spreading germs and viruses, cross-contamination of allergenic foods affecting children with allergies, and managing special dietary needs. Moreover, Lynch and Batal [33] cited a lack of available staff to manage multiple scenarios at mealtimes. Despite these very valid concerns, it is important to remember that children share toys and touch each other whilst at play, incidences which are just as likely to contribute to the transfer of germs, viruses or allergens. Hence, rather than restricting food-sharing practices, this paper posits that young children should still engage in food-sharing practices and family-style mealtimes at ECEC services, provided food hygiene standards can be maintained; this could be upheld by engaging children in education about hand washing prior to eating, and using tongs rather than touching shared food with their hands.

### 3.4. Food Sharing and Fussy Eating

Dealing with fussy eaters was another barrier to food sharing or family-style mealtimes identified by SNAC members. To overcome this barrier, participants described how they offered children autonomy in their food choices, reinforcing positive behaviour and persistence as strategies for overcoming fussy eating, all which can be incorporated into food sharing and family-style meal environments. Mimi, an educator, noted: “We kept offering. We didn’t modify the menu to suit certain palates beyond the general trying to find something that pleases everyone and taking it in turns to ensure favourites rotate through regularly. After a while, the kids just adapted to what was on offer… but I also think a lot about food refusal at first was simply not being comfortable with our environment yet and by respecting that little bit of autonomy we actually gave the kids the ability to choose to try the new things on offer with enthusiasm when they decided they were ready. Of course, we always give a lot of positive attention to kids when they are trying new things”. (Mimi, educator).

Positive reinforcement and persistence seemed to be successful for Mimi, and Jonine added further perspective by noting the positive impact peer role-modelling can have on children’s eating habits. Children are often influenced by their peers. Therefore, food sharing and family-style mealtimes offered further opportunities to overcome fussy eating issues. For example: “Basically with the children it’s the peer pressure but in a good way, if one’s eating it, one in we’re all in, we still do occasionally get that one child who will say I’m just not eating that, but the majority of children they see everybody else eating, it’s like sheep, they’ll just follow through and they’ll all have a go” (Jonine, Educator).

These examples illustrate the importance of providing self-service opportunities at family-style mealtimes, as this practice allows children to observe one another’s mealtime habits and food preferences and be influenced by these significant ‘others’. This concept has been described as “monkey see, monkey do”, in a study that reported children’s dietary intakes became similar to their peers’ over time, and as such, is a useful tool for combating fussy eating [55]. A study of sensory education sessions in Norwegian kindergartens also reported toddlers tended to positively influence each other’s food choices [56]. However, it is important that the food offered at mealtimes is nutritionally optimal for these peer role-modelling behaviours to be beneficial. 

### 3.5. Parental Influence on Food Sharing

ECEC services offer a vital and important service to working families, and in accordance with both the National Quality Standards and Early Years Learning Framework, they often seek feedback and input from parents around a number of issues, including food, nutrition and mealtimes. Many services ask parents to donate fruit for children’s morning tea, usually presented on platters for all to share. However, the director of a rural kindergarten revealed the following difficult situation: “At my kinder we have shared fruit in term 1, with all the children eating and most doing so enthusiastically (we thought); we did a survey to check parents feelings about doing it again in Term 2—as we ask for their help with cutting it up first thing in the morning—we got a majority of positives and a few did not respond, or speak to me personally. Then a Mum at the end of the last day told me that her child only wants to have his own fruit so he won’t be participating next term. I asked her if we could talk about it at the start of this term. I would like the child to be a part of it for his own sake as well as for everyone else’s—though obviously that aspect won’t matter to his Mum” (Lola). 

The SNAC members who responded demonstrated dismay at the parents’ intentions. Lola offered further explanation: “He will be coming [to kindy] but not sharing fruit—Mum says he doesn’t want to [share fruit] but she also says she doesn’t want to pay for fruit for other people’s kids—we have had one or two forget their fruit but I didn’t notice any grumblings, it wasn’t a serial thing for any child, and it seemed ok. Obviously not with this Mum!”.

Lola’s post suggested that this parent was concerned she would be subsidising other children’s morning teas, and subsequently requested that her child did not share with other children. However, this could be considered a somewhat superficial explanation for this parents’ request, and as her personal circumstances are unknown, it is worth considering her possible motivation. This particular kindergarten was located in a small, rural Victorian town, some 300 kilometres from the nearest major city. The cost of distributing fresh foods to rural locations is well-known and typically contributes to higher food costs for rural residents compared to metropolitan residents [57]. This parent’s unknown financial status may also be a factor, as food insecurity is reported to affect families at various income levels, not just those on very low incomes [58]. Whilst this parents’ decision is disappointing, there may be underlying factors that influenced her decision, highlighting the need for the implementation of public health initiatives to improve the variety and cost of fresh food available in rural locations [59]. Such initiatives are essential as a means of supporting food sharing, as evidence demonstrates children can learn compassion, exhibit kindness and care to others [60], whilst connecting with others and managing relationships [61], all contributing factors to social and emotional development in early childhood. Moreover, children who do not experience food-sharing environments, may have negative relationships with food and find it difficult to form social connections in later life [62].

## 4. Conclusions

Food sharing is a positive behaviour, contributing to children’s physical and psychological well-being. Eating food in a social context is an important aspect of food literacy, and a key to successful relationships with food throughout life. Therefore, teaching children to share food from a young age is an important stepping stone to developing their healthy relationships with food.

Through exploring the food-sharing practices in Australian ECEC services, we can conclude they are aware of the importance of food sharing and aimed to facilitate this wherever possible. However, a range of practical issues faced that deter services from food sharing need to be addressed. Firstly, concerns about food hygiene as food sharing (especially among very young children) involves possible contamination risks. Secondly, children’s age may influence decisions about food sharing, as it is perceived as difficult with children aged <3 years. Thirdly, ‘fussy eaters’ may refuse the food offered, thus impacting on the dynamics of food sharing. Finally, not all parents favour food sharing, and cultural differences in feeding practices may influence the decision to offer food-sharing opportunities.

Despite these barriers, and because the benefits of food sharing are becoming increasingly obvious across the lifespan, it is important to increase awareness among the global ECEC sector. We offer the following suggestions to overcome these obstacles.

- **Food hygiene**: Section 3.3 highlighted participants concerns about food hygiene as a barrier to family-style dining and/or food sharing in an ECEC setting. Children share toys and touch each other whilst playing, and it could be reasonably assumed that these habits also contribute to germ transfer and cross-contamination alongside food sharing. Whilst it is extremely important that all food preparation adheres to the highest standards, and food preparation staff are properly trained, we recommend that education about food sharing as an important aspect of food literacy should have greater impetus in early years’ education programs, as recommended elsewhere [28,29,30]. Trialing something as simple as a child-friendly ‘checklist’ could make the difference here, as the effectiveness of checklists in public health domains have been dramatic—for example, Atul Gawande’s work in the Checklist Manifesto [63]. We propose that this research area has not been applied specifically to child health behaviours and we plan to address this in further research.
- **Age of the child**: Section 3.2 highlighted differing opinions about the age of a child in relation to food sharing or self-serving. While food sharing may seem to become easier as children grow older, it is important to find ways for the youngest children to be provided with the agency to participate in food sharing [33]. For example, baby-led weaning could be introduced, whereby infants are offered chunks of softened vegetables in a family-style dining environment [51].
- **Fussy eaters**: Food sharing could be explored as a strategy to overcome fussy eating in young children, as discussed in Section 3.4. The “monkey see, monkey do” [56] concept supports the idea that children’s dietary intake becomes similar to their peers’ over time, so providing that the food provided is nutritionally sound and there are children present who are ‘good’ eaters, there is potential to improve fussy eating habits.
- **Parents**: Section 3.5 explored the influence that parents might play on the practice of food sharing in an ECEC setting, highlighting the importance of raising awareness among parents about the physical, social and emotional developmental benefits for their children [8]. ECEC staff should be supported to capacity-build and effectively address parent’s hygiene concerns, as recommended elsewhere [28,29,30].
- **Cultural aspects:** Accepting is an important aspect of day-to-day ECEC practices [54], and food sharing is a strategy where children can learn about different cultures from a young age. For example, Section 3.1 described practices of food sharing during Australian Harmony Week, where children enjoyed the foods and eating practices of different cultures, or on a child’s birthday, where food is shared according to their cultural background. Promoting such strategies may increase awareness and acceptance of cultural differences from a young age, and given the multicultural nature of modern Australian society, it is our duty to help children embrace the different fruits in our global bowl.

## 5. Strengths and Limitations

The qualitative, netnographic design of this research increased methodological rigour [41] and enabled the presentation of a holistic picture of the food environment at Australian ECEC services. The self-selected nature of the sample may be a limitation, as it is possible those SNAC members with a predisposed interest in healthy food and eating may have been more inclined to participate. However, generalising these results to the wider Australian population was not the intent of this study. 

In conclusion, we advocate for food sharing and family-style mealtime environments at ECEC services, and in other settings where young children eat, live and play, but recommend further research is conducted to further understand the impact such practices may have on the health and well-being of young children. If sharing is caring, then sharing foods on a daily basis could become a habit by which we demonstrate this care to those with whom we share life.

## Figures and Tables

**Table 1:** SNAC study data collection timepoint and method.

Date	Data Collected
1 August 2013–31 December 2014	Website: conversation threads, posts and comments
1 August 2013–31 December 2014	Participant observations during site visits; records of email and telephone conversations
September–December 2014	In-depth semi-structured interviews with individual members

**Table 2:** Themes around food sharing/family-style mealtimes.

Article/Theme	Question Asked	Posted By:	Section in Manuscript
Article: Family-style dining teaches kids to respond to hunger cues and fights obesity	What are your thoughts on this? Do you allow children to serve themselves? At what age do you think this is an appropriate practice?	Researcher	3.1, 3.2, 3.5
Article: Is lunchtime more than eating?	Are mealtimes at your service an opportunity to connect with children about healthy eating?	Researcher	3.1, 3.2, 3.3
Fussy eating or food refusal	Researcher: What is your practice if a child refuses lunch?	Researcher (prompted by member enquiry offline)	3.4
Harmony week	How do services use food to celebrate Harmony Week?	Researcher	3.1
Timing of mealtimes/progressive mealtimes	Initiated by a SNAC member enquiring about the timing of meals in Early Childhood Education and Care (ECEC) services	Posted by member	3.2

**Table 3:** Measures of trustworthiness.

General Term	Method Used	Description	Source
Transferability	Verisimilitude	Thick description of context, participants and research design	
	Purposive sampling	To ensure participants represented the context of the study design	Entrée [41] p. 6
Credibility	Triangulation	Combination of data collected using different methods at different time points	Detailed in the original SNAC study [39]
	Reflexivity [41]	Researcher recognises own role in the research	
Confirmability	Rigour [41]	Maintaining and recording an audit trail	
	Groundedness [41]	Clear links between presented data and theories to support study	
Dependability		Making and recording changes	
Authenticity	Praxis [41]: the extent to which practical action is aimed at social betterment	Participants were encouraged to engage with the community and take action when appropriate	
